# Imaging manifestations of small cell neuroendocrine carcinoma of the ureter

**DOI:** 10.1002/ccr3.6847

**Published:** 2023-01-23

**Authors:** Guiwu Chen, Xiong Huang, Wenqin Liu, Xiaomin Liao, Zhizhong He, Yuhuan Xie

**Affiliations:** ^1^ Department of Ultrasound, Affiliated Dongguan Hospital Southern Medical University, Dongguan People's Hospital Dongguan China; ^2^ Department of Ultrasound Dongguan Dongcheng Hospital Dongguan China; ^3^ Department of Pathology, Affiliated Dongguan Hospital Southern Medical University, Dongguan People's Hospital Dongguan China

**Keywords:** computed tomography, magnetic resonance urography, small cell neuroendocrine carcinoma, tumor of the urinary system, ultrasonography

## Abstract

Small cell neuroendocrine carcinoma (SCNEC) of the ureter is a rare malignant tumor originating from the metaplasia of urothelial cells. This report presents a case of ureteral SCNEC that was preliminarily disclosed by computed tomography; thereafter, transabdominal ultrasonography, transrectal ultrasonography, and magnetic resonance urography were performed to characterize the mass.

## Graphical abstract

Most small cell neuroendocrine carcinomas originating in the urinary system are located in the bladder and prostate: they rarely arise from the ureter. This report presents a case confirmed with ultrasonography, computed tomography, magnetic resonance urography, and pathological analysis.

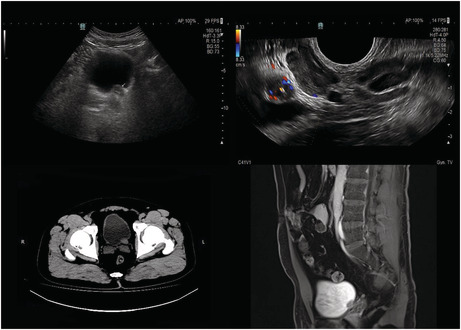

A 35‐year‐old man with a history of backache and hematuria over 1 week was admitted to our hospital. Transabdominal ultrasonography revealed dilation of the left renal collecting system and upper ureter due to blockage of the lower ureter by a hypoechoic mass. Coincidentally, the mass was located in the pelvic cavity next to the rectum. Transrectal ultrasonography was performed; a thickened lower ureter wall, whose structure was disordered and stiff, and additional details of the mass were obtained (Figure 1). Two days ago, abdominal computed tomography (CT) explored a soft tissue mass of the lower ureter that should be distinguished from tumors in other hospitals (Figure 2). Furthermore, magnetic resonance urography (MRU) suggested that the ureteral mass had a low signal filling defect, which was considered a carcinoma due to the lymph nodes being scattered around it (Figure 3). Ultimately, the patient underwent cystoscopy and surgical excision. Finally, pathological examination performed confirmed small cell neuroendocrine carcinoma (SCNEC) (Figure 4).

**FIGURE 1:**
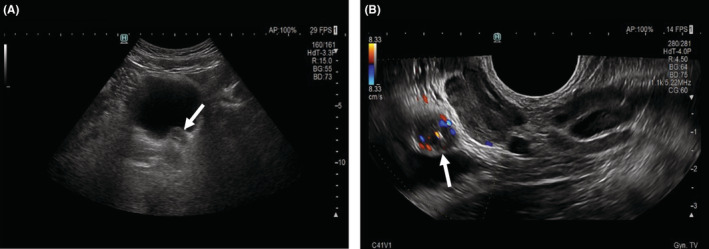
Ultrasonography of small cell neuroendocrine carcinoma of the ureter. (A) Transabdominal ultrasonography showed a hypoechoic mass located in the lower ureter (arrow) that was uniform on internal echo with an undefined boundary. (B) Transrectal ultrasonography showed that the blood flow of the mass was abundant (arrow), and the ureter wall around it was abnormal.

**FIGURE 2:**
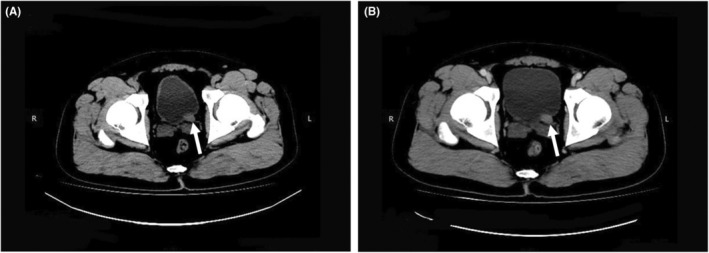
Computed tomography of small cell neuroendocrine carcinoma of the ureter. (A) Plain computed tomography scan showed a soft tissue mass of the lower ureter that was heterogeneous and well‐defined, which grew into the bladder cavity (arrow). (B) Enhanced computed tomography showed the soft tissue mass of the lower ureter that was slightly inhomogeneous enhancement (arrow).

**FIGURE 3:**
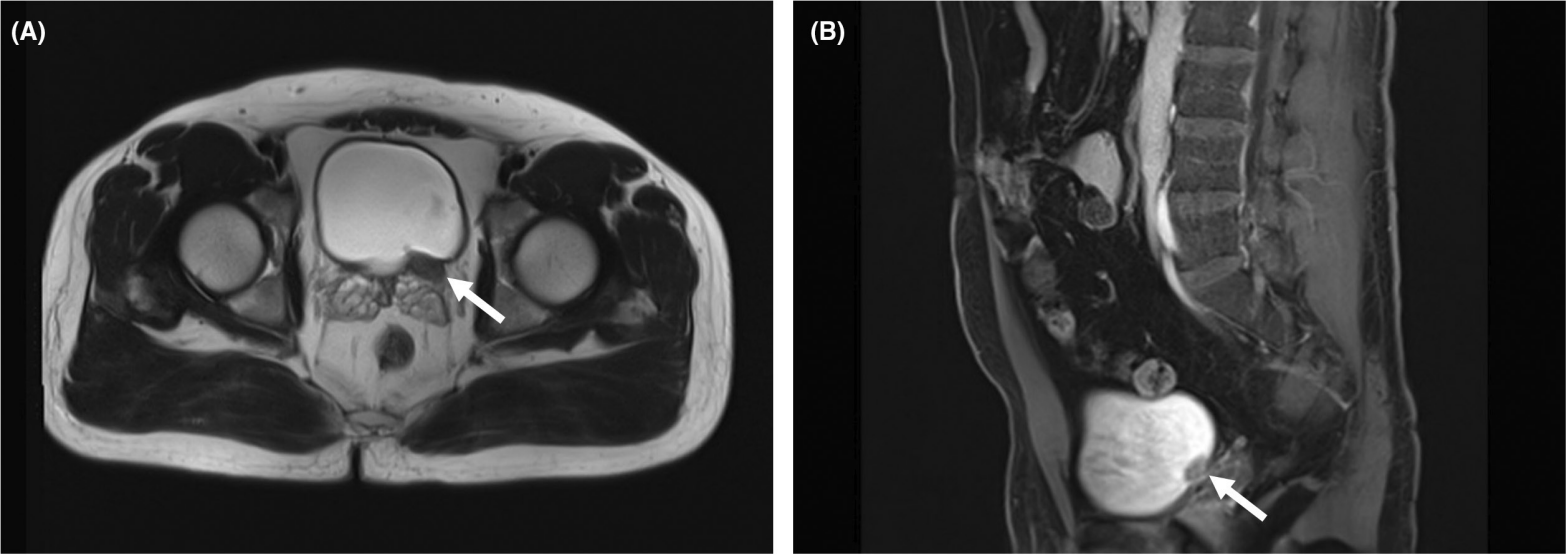
Magnetic resonance urography of small cell neuroendocrine carcinoma of the ureter. (A) The transverse section showed a mass that occurred in the left ureter (arrow). (B) The sagittal section showed the upper edge of the mass was cup‐shaped (arrow).

**FIGURE 4:**
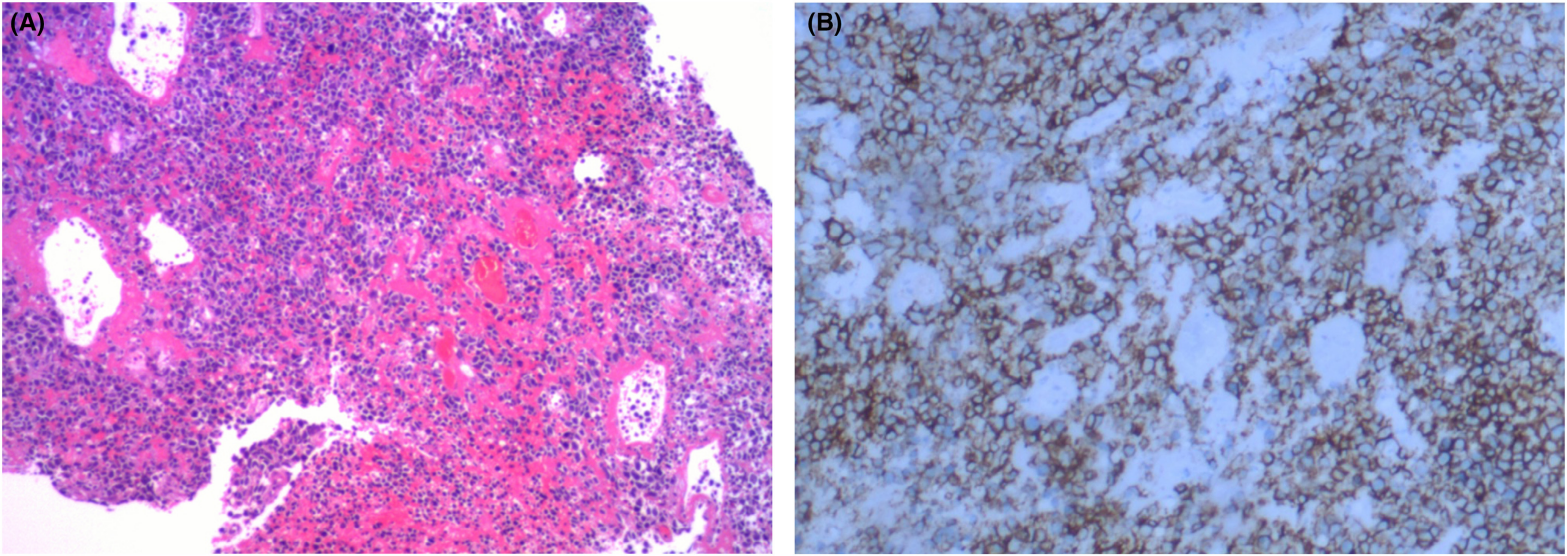
Pathology of small cell neuroendocrine carcinoma of the ureter. (A) Hematoxylin–eosin staining exposed the cells proliferated in a nest shape and grew infiltratively with obvious atypia, the nuclei of which were large and deeply stained. (B) Immunohistochemical staining findings were CD56‐positive, Syn‐positive, CK‐partially positive, TTF‐1 partially positive, CK20‐negative, GATA3‐negative, CgA‐negative, and Ki‐67 approximately 70% positive.

Small cell neuroendocrine carcinoma of the ureter is a highly malignant tumor originating from the metaplasia of urothelial cells with or without neuroendocrine function.^1^ Due to its rare nature, ureteral SCNEC is always misdiagnosed or escapes diagnosis in the early stage and has a poor prognosis in the later stage on imaging.^2^ However, SCNEC is usually detected by CT but rarely by ultrasonography and MRU, which could provide more features of ureteral SCNEC, including its composition, pattern, and blood flow. Imaging manifestations of inverted urothelial papilloma is a noninvasive endophytic urothelial neoplasm occurred in the renal pelvis, ureter, and urinary bladder, which shows iso‐intense on T1‐weighted images and either iso‐intense or slightly higher in intensity on T2‐weighted images.^3^ However, it is not uncommon to mistake SCNEC as other ureteral urothelial carcinomas depending on ultrasonography and MRU, especially high‐grade ureteral urothelial carcinoma. In conclusion, abdominal CT cannot be avoided and cystoscopy remains the diagnostic procedure of choice.^4^

## AUTHOR CONTRIBUTIONS

Guiwu Chen wrote the original draft of this clinical image and made the subsequent revisions. Xiong Huang participated in the computed tomography analysis and interpretation. Wenqin Liu and Zhizhong He participated in the ultrasonography and magnetic resonance urography analysis and interpretation. Xiaomin Liao participated in the pathology image analysis and interpretation. Yuhuan Xie assisted in the revision and supervised the overall production of this report.

## FUNDING INFORMATION

No funding was received for this study.

## CONFLICTS OF INTEREST

The authors declare that there is no conflict of interest.

## CONSENT

Written informed consent was obtained from the patient to publish this report in accordance with the journal's patient consent policy.

## Data Availability

The data used to support the findings of this study are available from the corresponding author upon request.
